# Difference between biomarkers of tibial bone marrow and adipose tissue

**DOI:** 10.1051/sicotj/2017022

**Published:** 2017-06-28

**Authors:** Ersin Kuyucu, Mehmet Erdil, Adnan Kara, Murat Bülbül

**Affiliations:** 1 Istanbul Medipol University, Orthopedics and Traumatology Clinic 34214 Istanbul Turkey

**Keywords:** Stem cells, Orthopedics, Bone marrow cells

## Abstract

*Background*: Stem cells, with their regeneration capacity, long-term viability, and differentiation characteristics, have indispensable biological properties. As described by Hauner and Grigoradis et al., mesenchymal stem cell originating from adipose or bone marrow can be differentiated into many tissues such as adipocyte, chondrocyte, myeloblast, and osteoblast. The aim of our study is to compare the use of adipose and tibial bone marrow derived stem cells for therapeutic purposes in orthopedic surgery, which has not been clearly evaluated in the literature to our knowledge and to also evaluate their use.

*Material and method*: Our study was performed between May 2014 and December 2016 in our clinic (Istanbul Medipol University, Department of Orthopedics and Traumatology) in 40 patients. Twelve patients were excluded. The ages of the 28 included patients ranged from 19 to 61 years, with a mean of 41.18 ± 13.39 years. The stem cell samples of these patients were analyzed by flow cytometry.

*Results*: Tibial bone marrow stem cells were used in 15 cases and the mean age was 49.33 ± 9.15. Adipose-derived stem cells were used in 13 patients and the mean age was 31.77 ± 11.25. None of the patients had any minor/major complication in the areas where stem cells were collected.

*Discussion*: Tibial-derived bone marrow has better results with regard to the complications, economic burden, and surgery time. Tibial-derived bone marrow harvesting and stem cell preparation time are one-fourth of the stem cell treatment prepared from adipose tissue and the surgical duration is shortened by 45 min.

*Conclusion*: If stem cell use is the preference of the surgeon, we have found that the tibial-derived stem cell system is more advantageous for ease of acquisition, cost analysis, and surgical time.

## Background

Nowadays, cellular therapy with stem cells is being used increasingly in orthopedic surgery as well as in many other fields. Stem cells, with their regeneration capacity, long-term viability, and differentiation characteristics, have indispensable biological properties [1]. Stem cells are multipotent cells that exist unmodified in tissues such as amniotic cord, bone marrow, adipose tissue, and the central nervous system. In the course of damage to their located tissues, their differentiation helps to heal damaged tissue and this is their indispensable feature [2]. As described by Hauner and Grigoradis et al., mesenchymal stem cell originating from adipose or bone marrow can be differentiated into many tissues such as adipocyte, chondrocyte, myeloblast, and osteoblast [3, 4].

Non-embryonic stem cells are often obtained from adipose tissue or bone marrow, although they can be obtained from many tissues such as blood, synovial membrane, skin, or muscle tissue [5]. Bone marrow stem cells can be obtained from the pelvis, femur, or tibia. Clinical use has been on the rise since the early 2000s. The use of it if necessary via a scaffold matrix without the need for a secondary operation is a significant advantage. The acquisition of stem cells from the adipose tissue has been particularly noted with the study performed by Zuk et al. [6]. Although it is a minimally invasive surgery with advanced techniques there are minor local complications such as contour irregularity, hyperpigmentation, and necrosis and there are also major mortal complications such as sepsis, fat embolism, and pulmonary embolism [7].

The aim of our study is to compare the use of adipose and tibial bone marrow derived stem cells for therapeutic purposes in orthopedic surgery, which has not been clearly evaluated in the literature as much as we know before and to evaluate the use details.

## Material and method

Our study was performed between May 2014 and December 2016 in our clinic (Istanbul Medipol University, Department of Orthopedics and Traumatology) in 28 patients, 60.7% (*n* = 17) female, 39.3% (*n* = 11) male, who were operated due to nonunion, gonarthrosis, osteochondral defect, and tibial or adipose stem cell used and retrospectively analyzed. The study was carried out in hospital and stem cell laboratory based on computer registered digital data.

The ages of the cases ranged from 19 to 61 years, with a mean of 41.18 ± 13.39 years. When the patients treated with tibial stem cells were examined: seven patients had Kellgren-Lawrence stage 2 gonarthrosis, six patients had Kellgren-Lawrence grade 3 gonarthrosis, four patients had talus osteochondral defects, and two patients had nonunion. The stem cell samples of these patients were analyzed by flow cytometry, and 15 of 19 patients who were suited to the study and adequately analyzed were enrolled in the study. When the patients treated with adipose stem cells were examined; eight patients had a tibial nonunion, five patients had a femoral nonunion, five patients had Lawrence grade 2 gonarthrosis, and three patients had Lawrence grade 3 gonarthrosis. The stem cell samples of these patients were analyzed by flow cytometry, and 13 of 21 patients who were suited to the study and adequately analyzed were enrolled in the study.

## Harvesting stem cells from tibial bone marrow

**Figure 1.**
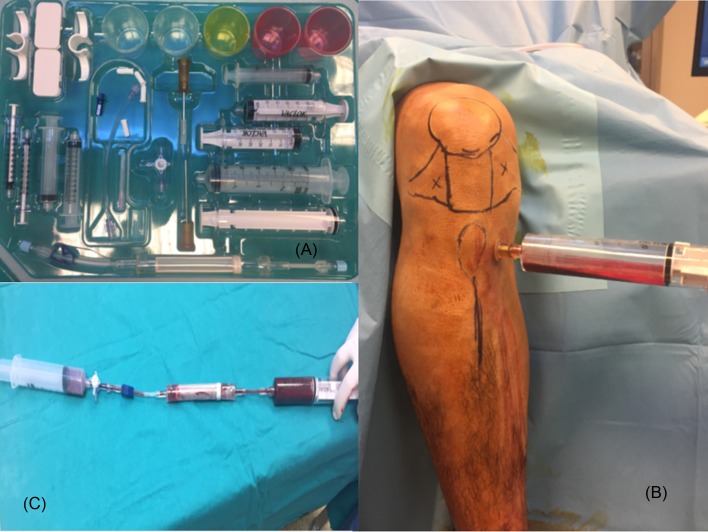
(A) Tibial bone marrow system, (B) harvesting the bone marrow sample, (C) preparing the sample.

After the appropriate anesthesia and covering procedures were carried out, a mini-incision was made with a 15-point bistula about 1 cm medial and 1 cm distal to the tibial tuberosity without tourniquet application. The proximal tibial metaphysis was entered with a system drill, an injector previously loaded with 5 cc citrate applied into the drill cannula and 55 cc liquid aspirated. A total of 60 cc aspirate was first filtered through the system and the resulting clots were cleared and the resulting aspirate was centrifuged for 15 min at a spin rate of 2800 rpm and a second spin of 3800 rpm and a total of 4 cc stem cells obtained. One cubic centimeter was used for flow cytometry and 3 cc was used during the surgical procedure. The Magellan (Biologic Therapies) system stem cell set was used for all patients (Figure 1). The procedure was performed by two different surgeons (M.B-E.K) experienced in obtaining stem cells from the tibial bone marrow. The preparation time is a standard 15 minutes.

## Harvesting stem cells from adipose tissue

While the patients were under general anesthesia, cells were obtained from the fat tissue excreted through umbilicus by using a body jet liposuction technique in the umbilical region. This procedure was done by a plastic surgeon. Prior to liposuction, 1 cc (2.5 mg) of adrenaline and 10 cc of lidocaine diluted with isotonic solution totally 400 mL solution were injected into the areas of fat collection. Then, about 300 mL of fat was collected with liposuction cannulas. After 10 min of washing with a centrifuge, 30 min of incubation was performed and then the sample centrifuged again for 15 min. The preparation time is a standard 60 min.

## Flow cytometry

The sample was diluted with 10 mL of Dulbecco’s Modified Eagle’s Medium (DMEM) solution before the cytometry. Cells were collected under centrifugation at 400 mg/5 min. Stem cell specific CD14-31-45-166-34-44-140b-13-117-105-90-29-73 analyses were performed.

## Statistical review

The NCSS (Number Cruncher Statistical System) 2007 (Kaysville, Utah, USA) program was used for statistical analysis. When study data were evaluated in addition to descriptive statistical methods (mean, standard deviation, median, frequency, ratio, minimum, maximum) Student’s *t* test was used for two group comparisons of normal distributions in the comparison of quantitative data, and Mann-Whitney *U* test was used for two group comparisons of non-normal distributions. A comparison of qualitative data was made using the Yates’ Continuity Correction test (Yates’ corrected chi-square). Significance was assessed at *p* < 0.05 level.

## Results

Our study was carried out between May 2014 and December 2016 with 28 patients, 60.7% (*n* = 17) female and 39.3% (*n* = 11) male, who required stem cell therapy at the Istanbul Medipol University. The ages of the cases ranged from 19 to 61 years with a mean of 41.18 ± 13.39 years, 17 (60.7%) women and 11 (39.3%) men. Tibial bone marrow stem cells were used in 15 cases and the mean age was 49.33 ± 9.15. Adipose-derived stem cells were used in 13 patients and the mean age was 31.77 ± 11.25. None of the patients had any minor/major complication in the areas where stem cells were collected.

The time of sampling (*p* = 0.001), duration of stem cell preparation (*p* = 0.001), and total time (*p* = 0.001) were found to be statistically significantly higher in the adipose tissue stem cell group (*p* < 0.01).

CD14 measurements were statistically significantly higher in the tibial bone marrow stem cell group (*p* = 0.001, *p* < 0.05).

Measurements of CD31 (*p* = 0.001), CD45 (*p* = 0.033), and CD44 (*p* = 0.001) were statistically significant in the tibial bone marrow stem cell group (*p* < 0.05).

Expression of CD73, CD90, and CD105 in both methods was found to be 99% and over (*p* > 0.05)

## Discussion

In our study we evaluated the stem cell treated patients according to the stem cell resources as bone marrow or adipose tissue, which is an increasingly used method in the 21st century and has various indications in orthopedic surgery. We found that the tibial bone marrow-derived stem cell was both easier and quicker to prepare and had similar flow cytometry results. We also found that hematopoietic-derived markers, such as CD31-44-14-45, are more abundant in the stem cell from the tibial bone marrow.

Mesenchymal stem cells, which can be obtained from many tissues such as blood, muscle, synovial membrane, bone marrow, and fat tissue, are characterized by their ability of differentiation to various tissues such as osteocytes, adipocytes, tenocytes, and chondrocytes [3, 4, 6]. Because of these important features they have been used in orthopedic surgery in the treatment of osteoarthritis, nonunion, cartilage damage, tendon injury, especially in the last decade [7, 8]. Cui et al.’s meta-analysis showed that mesenchymal stem cell application in osteoarthritis patients had a continuous effect for up to 24 months [9]. Jin et al also described successful results in treating cartilage damage with bone marrow-derived mesenchymal stem cells [10].

Stem cells in addition to differentiation, including many bioactive molecules, which also fulfill many important features such as tissue repair, inflammation suppression, apoptosis inhibition, immunomodulation, and angiogenesis [11, 12]. CD31, known as platelet endothelial cell adhesion molecule (PECAM-1), is primarily responsible for angiogenesis and integrin activity. Woodfin and colleagues have reported that PECAM-1 (CD31) is involved in many cell walls such as leukocytes, T-lymphocytes, neutrophils, monocytes, and is involved in angiogenesis, immunomodulation, and cell signaling [13]. When we compared stem cell values derived from adipose and tibial bone marrow, we found that CD31 expression in tibial-derived stem cells was higher at a statistically significant level. Another important receptor, CD44, is involved in many cell types and acts primarily as a receptor for hyaluronic acid, collagen, and osteopontin [14]. It increases the migration especially of hematopoietic and mesenchymal stem cells in bone marrow. CD45, known as the leukocyte common antigen, is primarily responsible for cell growth, mitotic activity, and cell differentiation [14, 15]. In our analyses, the stem cell, in particular from the tibial bone marrow was found higher at a statistically significant level. We know that the tibial bone marrow cell ratio is less than the iliac bone marrow cell ratio, however it is not important when you harvest the bone marrow systematically and you can get the appropriate bone marrow for the treatment [16].

Especially when cost analysis is one of the most important problems today, liposuction brings an additional burden. LaBove and Davison reported that, minor plastic surgery, such as liposuction, costs an average of $1200 [17]. Requiring a plastic surgeon as well as an orthopedic surgeon is also a difficult problem. Tibial-derived stem cell application does not add any additional cost and it does not depend on other surgeons thus making this application very advantageous.

When compared with the additional surgery time, tibial-derived bone marrow harvesting and stem cell preparation time are one-fourth of the stem cell treatment time for adipose tissue and the surgical duration is shortened by 45 min, which reduces both the exposure of the patient to anesthetic drugs and reduces the complications that are directly proportional to the increase in surgical time, such as infection of the surgical field and embolism [18].

The strengths of our study are the comparison of two different methods of obtaining stem cells, which are frequently used in orthopedic surgery. The most important limitation of our study is that it does include clinical results which is not the aim of this study. The small number of analyses is our other limitation (Table 1).

**Table 1.** Comparison of stem cell values derived from adipose tissue and tibial bone marrow.

	Total	Adipose tissue stem cell	Tibial bone marrow stem cell	a*p*
		Min-Max	Min-Max	
Age	19–61 (43)	19–56 (31)	34–61 (51)	0.001**
	41.18 ± 13.39	31.77 ± 11.25	49.33 ± 9.15	
Sex	Kadın	8 (47.1)	9 (52.9)	b1.000
	Erkek	5 (45.5)	6 (54.5)	
Sample time	2–33 (3.5)	14–33 (21)	2–4 (2)	0.001**
	11.00 ± 9.82	20.77 ± 4.87	2.53 ± 0.64	
Stem cell preparation time	15–60 (15)	60–60 (60)	15–15 (15)	0.001**
	35.89 ± 22.85	60.00 ± 0	15.00 ± 0	
Total time	17–93 (18.5)	74–93 (81)	17–19 (17)	0.001**
	46.89 ± 32.28	80.77 ± 4.87	17.53 ± 0.64	
Hemogram	3.67–8.45 (6.98)	3.6–8.13 (5.56)	6.45–8.45 (7.3)	0.003**
	6.61 ± 1.39	5.71 ± 1.50	7.39 ± 0.63	
Hematocrit	12.4–22.5 (18.75)	12.4–22.5 (16.43)	17.2–20.8 (19.2)	0.080
	18.25 ± 2.54	17.28 ± 3.40	19.09 ± 0.95	
Viability	99.03–99.89(99.73)	99.38–99.89(99.79)	99.03–99.72(99.59)	0.070
	99.68 ± 0.35	99.72 ± 0.45	99.52 ± 0.33	
CD14	0.11–10.02 (4.76)	0.11–8.32 (3.12)	2.23–10.02 (6.83)	0.001**
	4.91 ± 2.89	3.14 ± 2.51	6.45 ± 2.29	
CD31	10.89–90.45 (37.56)	11.34–58.77 (21.43)	10.89–90.45 (50.43)	0.001**
	39.34 ± 21.97	24.48 ± 12.88	52.23 ± 20.14	
CD45	10.07–98.84 (35.64)	13.25–98.84 (27.26)	10.07–71.7 (45.4)	0.033*
	40.52 ± 21.21	33.56 ± 23.02	46.56 ± 18.14	
CD44	11.09–97.45 (38.18)	11.09–97.45 (16.5)	26.88–88.6 (70.2)	0.001**
	44.92 ± 28.70	24.26 ± 22.90	62.82 ± 19.99	
CD34	0.01–0.01 (0.01)	0.01–0.01 (0.01)	0.01–0.01 (0.01)	1.000
	0.01 ± 0	0.01 ± 0	0.01 ± 0	
CD166	0.01–3.54 (0.01)	0.01–3.54 (0.71)	0.01–0.02 (0.01)	0.001**
	0.5 ± 1.00	1.12 ± 1.27	0.01 ± 0	
CD13	0.01–14.9 (0.06)	0.01–14.9 (3.33)	0.01–2.64 (0.01)	0.001**
	1.8 ± 3.07	3.65 ± 3.71	0.2 ± 0.68	
CD140b	0.01–1.92 (0.02)	0.01–1.92 (0.01)	0.01–1.64 (0.04)	0.058
	0.17 ± 0.46	0.18 ± 0.53	0.16 ± 0.41	
CD117	0.01–0.01 (0.01)	0.01–0.01 (0.01)	0.01–0.01 (0.01)	1.000
	0.01 ± 0	0.01 ± 0	0.01 ± 0	
CD105	0.01–8.36 (0.11)	0.01–8.36 (0.01)	0.01–4.68 (0.23)	0.080
	1.02 ± 1.86	1.05 ± 2.33	1.00 ± 1.43	
CD90	0.01–3.75 (0.03)	0.01–3.75 (0.03)	0.01–0.26 (0.05)	0.717
	0.35 ± 0.87	0.65 ± 1.23	0.09 ± 0.09	
CD29	0.01–0.01 (0.01)	0.01–0.01 (0.01)	0.01–0.01 (0.01)	1.000
	0.01 ± 0	0.01 ± 0	0.01 ± 0	
CD73	0.01–4.71 (0.01)	0.01–0.01 (0.01)	0.01–4.71 (1.01)	0.015*
	0.70 ± 1.33	0.01 ± 0	1.29 ± 1.61	

a *p* value.

b No sex meaning.

* Statistically strong meaning.

** Statistically very strong meaning.

## Conclusion

If stem cell use is the preferred therapeutic preference of the surgeon, we have found that the tibial-derived stem cell system is more advantageous for ease of acquisition, cost analysis, and shorter operative time.

**Abbreviations**
PECAM: Platelet endothelial cell adhesion molecule
CD: Cluster of differentiation

## Ethics approval and consent to participate

This study was unanimously approved by the Ethics Committee of Medipol University Local Ethics Committee with Decision Number 12001.2017.

## Conflict of interest

The authors declare that they have no conflict of interest.

## Authors’ contributions

EK organized the study and writing. ME and AK carried out the writing. MB organized the surgeries.
